# Chloroform in Sympathetic Vomiting

**Published:** 1853-12

**Authors:** Thos. Inman


### *Chloroform in Sympathetic Vomiting.*

BY DR. THOS. INMAN.

[For this symptom two classes of remedies are generally resorted to—stimuli, or direct sedatives. One of the most valuable of these is creasote, but on account of its many disagreable qualities, Dr. Inman suggests chloroform, in the dose of three or four drops, well shaken up with water, to be used in its place. He says:]

I do not know whether the suggestion is new ; it was forced upon me by circumstances. A friend came to visit us across the sea, and suffered urgently from sea-sickness, which continued long after her arrival, to such an extent, that any motion of the body produced vomiting. Not having anything else in the house but chloroform, I gave some of that, and was gratified to find that its success was immediate. The next case was one occurring in the practice of a friend, where the vomiting had been kept up incessantly for three days, and where creasote had been unavailing. The vomiting was partly due to an overflow of bile, and partly to pregnancy: it continued, however, after the flow of bile had ceased, and was beginning to weaken the patient materially. The first dose of chloroform (five drops) checked the vomiting for six hours ; there was then a slight repetition of the sickness, which, however, disappeared entirely after another dose.

The next case was one of vomiting from disorder of the liver. The first dose put a stop to the sickness, and had not to be repeated.

My next experience was in the case of the lady I first mentioned, who found it useful in preventing sea-sickness.

I have induced some of my friends also to try it, and they give an equally favorable report concerning it.

Its chief advantages over creasote are, its pleasant taste in the mouth as it goes down, and its not unpleasant flavor if it comes up again.—The only point requiring attention is, that the mixture must be well agitated immediately before being taken, as the chloroform rapidly falls to the bottom of the spoon or glass.—*Med. Times and Gazette.*

